# Topographical distribution and morphology of NADPH-diaphorase-stained neurons in the human claustrum

**DOI:** 10.3389/fnsys.2014.00096

**Published:** 2014-05-27

**Authors:** Dimka V. Hinova-Palova, Lawrence Edelstein, Boycho Landzhov, Minko Minkov, Lina Malinova, Stanislav Hristov, Frank J. Denaro, Alexandar Alexandrov, Teodora Kiriakova, Ilina Brainova, Adrian Paloff, Wladimir Ovtscharoff

**Affiliations:** ^1^Department of Anatomy, Histology, and Embryology, Medical UniversitySofia, Bulgaria; ^2^Medimark CorporationDel Mar, CA, USA; ^3^Department of Anatomy and Histology, Medical UniversityVarna, Bulgaria; ^4^Department of Forensic Medicine and Deontology, Medical UniversitySofia, Bulgaria; ^5^Department of Biology, Morgan State UniversityBaltimore, MD, USA

**Keywords:** human claustrum, NADPH-diaphorase, nitric oxide, nitric oxide synthase, projection neurons, interneurons

## Abstract

We studied the topographical distribution and morphological characteristics of NADPH-diaphorase-positive neurons and fibers in the human claustrum. These neurons were seen to be heterogeneously distributed throughout the claustrum. Taking into account the size and shape of stained perikarya as well as dendritic and axonal characteristics, Nicotinamide adenine dinucleotide phosphate-diaphorase (NADPHd)-positive neurons were categorized by diameter into three types: large, medium and small. Large neurons ranged from 25 to 35 μm in diameter and typically displayed elliptical or multipolar cell bodies. Medium neurons ranged from 20 to 25 μm in diameter and displayed multipolar, bipolar and irregular cell bodies. Small neurons ranged from 14 to 20 μm in diameter and most often displayed oval or elliptical cell bodies. Based on dendritic characteristics, these neurons were divided into spiny and aspiny subtypes. Our findings reveal two populations of NADPHd-positive neurons in the human claustrum—one comprised of large and medium cells consistent with a projection neuron phenotype, the other represented by small cells resembling the interneuron phenotype as defined by previous Golgi impregnation studies.

## Introduction

The claustrum is a telencephalic structure present in nearly all mammalian brains (Guirado et al., 2003; Real et al., 2003; Ashwell et al., 2004; Edelstein and Denaro, 2004). In humans, it is situated between the insular cortex and putamen, and bordered by the external and extreme capsules. The claustrum was first depicted by the noted French physician/anatomist Felix Vicq d' Azyr in his historic treatise (Vicq d'Azyr, 1786) and described as: “*Tractus cortical très délié qui se trouve entre le sillon de Sylvius et les corps striés* [*‘Separated cortical tract between the Sylvian fissure and the corpus striatum’*]” It is generally believed that the German physiologist Karl Burdach first ascribed the name “claustrum” to this nucleus (Burdach, 1822). The size of the claustrum varies by species (Berlucchi, 1927; Brockhaus, 1940; Macchi, 1948; Rae, 1954; Berke, 1960; Narkiewicz, 1964; Filimonoff, 1966; Druga, 1974, 1975; Zilles et al., 1980; Paxinos and Watson, 1998; Kowianski et al., 2004). The gross anatomical subdivisions of the claustrum are generally accepted as being dorsal (insular) and ventral (endopiriform) (Guirado et al., 2003; Ashwell et al., 2004; Edelstein and Denaro, 2004). Morys et al. (1996) further divided the claustrum into four parts: dorsal, orbital, temporal, and paraamygdalar. Namavar et al. (2005) distinguished three parts in the dorsoventral plane; cap, dorsal, and ventral.

The dorsal claustrum is connected with the neocortex (Narkiewicz, 1964; Druga, 1966a,b, 1968, 1975; Narkiewicz, 1972; Norita, 1977; Kunzle, 1978; Riche and Lanoir, 1978; Edelstein and Denaro, 1979, 1980; Olsen and Graybiel, 1980; Neal et al., 1986; Sloniewski et al., 1986a,b; Tanne-Gariepy et al., 2002). The ventral claustrum is situated just beneath the piriform cortex, well-interconnected with prepiriform and entorhinal cortices (Druga, 1971; Witter et al., 1988; Dinopoulos et al., 1992). Braak and Braak (1982) distinguished five types of neurons in the human claustrum: Type I representing spiny neurons varying in size and shape, Type II are large aspiny neurons, Type III are large aspiny neurons devoid of pigment deposits, Type IV are small pigment-laden aspiny neurons, and Type V are small aspiny neurons devoid of lipofuscin granules. The functional significance of the claustrum continues to be the subject of debate (Edelstein and Denaro, 2004; Crick and Koch, 2005; Smythies et al., 2012, 2014a,b). A recent comprehensive review by Sherk (2014) on the physiology of the claustrum offers considerable insight into this structures complex interplay with both sensory and motor cortices, while discussing hypotheses as to its function.

Nicotinamide adenine dinucleotide phosphate-diaphorase (NADPHd)-positive neurons and fibers are present in many parts of the nervous system in a variety of species, including humans (Mizukawa et al., 1989; Mizukawa, 1990; Vincent and Kimura, 1992; Druga and Syka, 1993; Valtschanoff et al., 1993; Yan et al., 1996; Hinova-Palova et al., 1997; Paloff and Hinova-Palova, 1998; Moreno-Lopez et al., 1998; Saxon and Beitz, 2000; Lysakowski and Singer, 2000; Holstein et al., 2001; Martinelli et al., 2002; Papantchev et al., 2005, 2006; Edelstein et al., 2012a,b). Histochemical mapping studies of the mammalian brain reveal either neuronal nitric oxide synthase (NOS) or NADPHd activity in the claustrum (Vincent and Kimura, 1992; Paloff et al., 1994; Rodrigo et al., 1994; Switka et al., 1994; Hinova-Palova et al., 1997, 2013; Paloff and Hinova-Palova, 1998; Paxinos and Watson, 1998; Vincent, 2000; Edelstein et al., 2012a,b).

Under specific fixation conditions, NADPHd is routinely used as a histochemical marker for NOS (Mizukawa et al., 1989; Dawson et al., 1991; Hope et al., 1991; Bredt and Snyder, 1992; Vincent and Hope, 1992; Vincent and Kimura, 1992; Druga and Syka, 1993; Paloff et al., 1994; Switka et al., 1994). According to Matsumoto et al. (1993), paraformaldehyde adversely impacts NADPHd labeling, thus negating any observable correlation between NADPHd staining and NOS immunocytochemistry (Dun et al., 1992). In contrast to these studies, efforts by Terenghi et al. (1993) in humans and rats, as well as those by Artero et al. (1995) in the crested newt, have demonstrated a good correlation in similarly fixed tissue.

The demonstration that NADPHd staining is due to the activity of NOS (Hope et al., 1991) quickly facilitated the detailed anatomical analysis of NO-producing cells throughout the nervous system (Vincent and Hope, 1992; Vincent and Kimura, 1992). The direct relationship between NADPHd staining and NOS expression has been well-documented (Bredt et al., 1991; Dawson et al., 1991). The absence of neuronal expression of NADPHd and NOS activity in knockout mice lacking NOS provided definitive evidence for the specificity of this simple histochemical procedure (Huang et al., 1993). The results obtained with NADPHd histochemistry have been confirmed and extended using antibodies against the various NOS isoforms (Bredt et al., 1990) as well as with *in situ* hybridization (Bredt et al., 1991). Of particular importance has been the description of alternatively-spliced forms of NOS expressed in certain brain regions (Brenman et al., 1997; Eliasson et al., 1997). Freire et al. (2004, 2005, 2010) used NADPHd histochemistry to study cortical fields in several different animals, including barrel cortical fields in the rat and mouse, and V1, V2, V3 in the common agouti.

Recently, there has been an increasing focus on nitric oxide (NO), which participates in many physiological and pathological processes. In the brain, NO is an important messenger molecule involved in such diverse functions as transsynaptic transmission, neuronal development, plasticity, the release of neurotransmitters, as well as long-term synaptic modulation (Garthwaite, 1991; Schuman and Madison, 1991; Sibuki and Okada, 1991; Lorrian and Hull, 1993; Zhuo et al., 1993; Bredt and Snyder, 1994). With respect to the peripheral nervous system, NO serves as a transmitter in non-adrenergic and non-cholinergic neurons (Bult et al., 1990). NO is also produced in the cardiovascular, renal and lymphatic systems, thus playing an important role in hemodynamics, and vasodilatation (Palmer et al., 1987; Nathan and Nibs, 1991; Nathan, 1992; Romero et al., 1992). It also plays a role in various neurodegenerative disorders such as Parkinson's disease, Alzheimer's disease, and Huntington's disease (Ferrante et al., 1985, 1987; Koh et al., 1986; Boegman and Parent, 1988; Halliwell, 1989; Dawson et al., 1991; Mufson and Brandabur, 1994; Hunot et al., 1996; Tao et al., 1999; Freire et al., 2007). According to Aoki et al. (1995), NO controls a number of critical physiological processes. In this regard its actions run the gamut, from signal transduction to apoptosis, with its synthesis catalyzed by NOS (Bredt et al., 1990).

NOS has been localized to a variety of other organs in rats and humans (Bredt and Snyder, 1990; Bredt et al., 1990; Dun et al., 1992; Springall et al., 1992), including the central nervous system (CNS) of the crested newt (Artero et al., 1995). Using immunocytochemical procedures, Rodrigo et al. (1994) detailed the mapping of NOS in the adult rat brain.

Mizukawa et al. (1989) and Vincent and Kimura (1992) did not report on the topographical distribution of NO immunoreactive neurons in the claustrum, in the context of their CNS studies. The existence of NOS neurons in the claustrum has previously been noted, but in limited scope (Paloff et al., 1994; Rodrigo et al., 1994; Switka et al., 1994). To the best of our knowledge, ours is the first detailed assessment of the topographical distribution and morphological characteristics of NADPHd-positive neurons and fibers in the human claustrum.

The investigations of our laboratory have been focused on the cytoarchitecture, ultrastructure, immunocytochemistry, and connections of the claustrum for over 30 years (Hinova-Palova et al., 1979, 1980a,b, 1988, 1997, 2001, 2007, 2008, 2012a,b; Hinova-Palova, 1981, 1986; Hinova-Palova and Usunoff, 1981; Hinova-Palova and Christova, 1988a,b; Hinova-Palova and Braak, 1993; Edelstein et al., 2011a,b, 2012a,b). The internal complexity and intricate cortical interrelationships of this broad and sheet-like telencephalic mass comes as no surprise, given its diverse neuronal composition and intrinsic functional heterogeneity. Its topographical and functional relations with most if not all cortical and adjacent brain areas make it an especially interesting and challenging area for investigation (Edelstein and Denaro, 2004; Smythies et al., 2012, 2014a,b). It is the intention of this study to describe and depict the topographical distribution and morphology of NADPHd-positive neurons and fibers in the human claustrum.

## Materials and methods

Brains were removed at 4–10 h post-mortem from four male patients (28, 42, 58, and 61 years of age) and three female patients (40, 52, and 68 years of age) with no known neurological disorders. The brains were blunt cut in the coronal plane into 1–2 cm slabs, then fixed for 2 days under gentle agitation in a mixture of 4% paraformaldehyde, 2% glutaraldehyde, 1% picric acid, and 10% glucose.

Next, the slabs were blocked to the area of interest, washed in 0.01 M phosphate-buffered saline (PBS) and sectioned at 40 μm on a freezing microtome (Reichert-Jung). All sections were treated with sodium borohydride for 45 min followed by three consecutive 2-min rinses in 0.01 M PBS. The slices were then incubated in a solution containing 0.2 mg/ml nitro blue tetrazolium chloride (NBT), 1 mg/ml NADPH tetrasodium salt and 0.5% Triton X-100 diluted in 0.1 M Tris-HCl buffer with pH 7.4 at 37°C for 30–60 min. The reaction was completed with 0.1 M Tris-HCl, pH 7.6. Afterwards, sections were rinsed three times for 5 min each in the same phosphate buffer, air-dried for 24 h and cover-slipped with Entellan (Merck Millipore). NADPHd staining was visualized as a blue reaction product within the neuronal cytoplasm.

For quantitative analysis, 40 slides containing sections of NADPHd-positive neurons were examined using an image analyzer (CUE-2, Olympus) and a 40× objective. The analysis began with capturing and storing images of the area of interest. Afterwards, we performed standard planar morphometry and linear analysis (i.e., line length and width). Next, we measured the maximum diameter of 700 neurons, and the cells were then divided into groups. A mean was then calculated for the minimum and maximum diameter of all neurons in each group. A Mann-Whitney analysis was performed to determine whether the special distribution and size differences were statistically significant. Subsequent to quantitative analysis, explanatory markers were added to all images using Adobe Photoshop 7.0.

## Results

**Figure 1:**
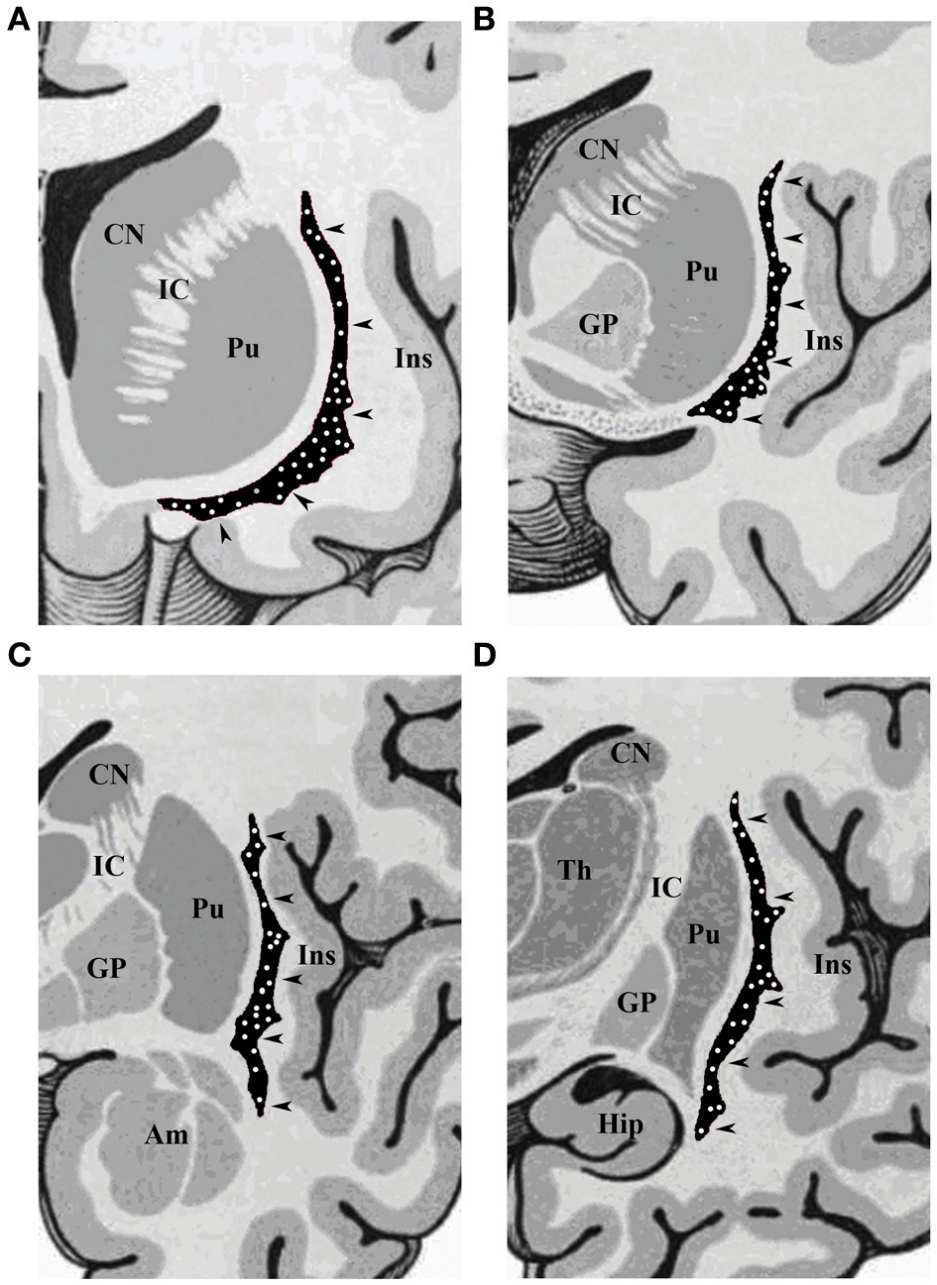
**Schematic drawings of coronal sections of human brain, from rostral to caudal through the (A) olfactory tract, (B) anterior commissure, (C) amygdaloid body, and (D) hippocampus**. CN, caudate nucleus; IC, internal capsule; Pu, putamen; Ins, insular cortex; GP, globus pallidus; Am, amygdala; Hip, hippocampus; Th, thalamus. The arrowheads indicate the claustrum, with white dots depicting the topographical distribution of NADPHd-positive neurons.

**Figure 2:**
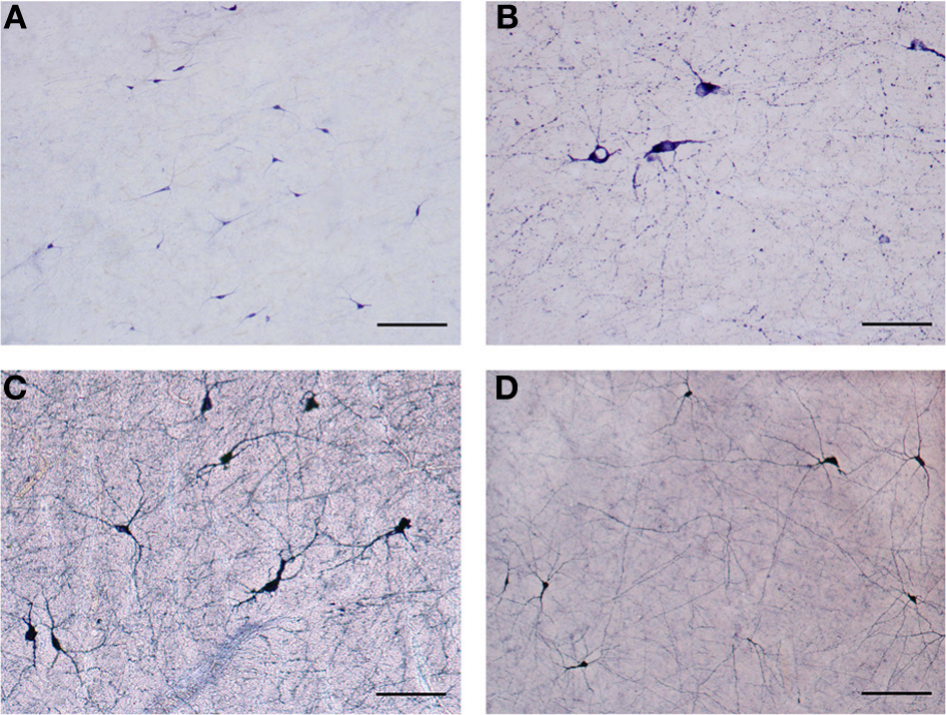
**(A)** Low magnification of NADPHd-positive neurons in the claustrum. Note the heterogeneity of size and shape. Scale bar = 300 μm. **(B)** NADPHd-positive neurons and fibers in the caudal pole of the claustrum. Cell bodies are few, but the fibers and their varicose nature are quite evident. Scale bar = 100 μm. **(C)** NADPHd-positive neurons in the ventral claustrum. Scale bar = 100 μm. **(D)** NADPHd-positive neurons and fibers in the caudal third of the ventral claustrum. Scale bar = 200 μm.

**Figure 3:**
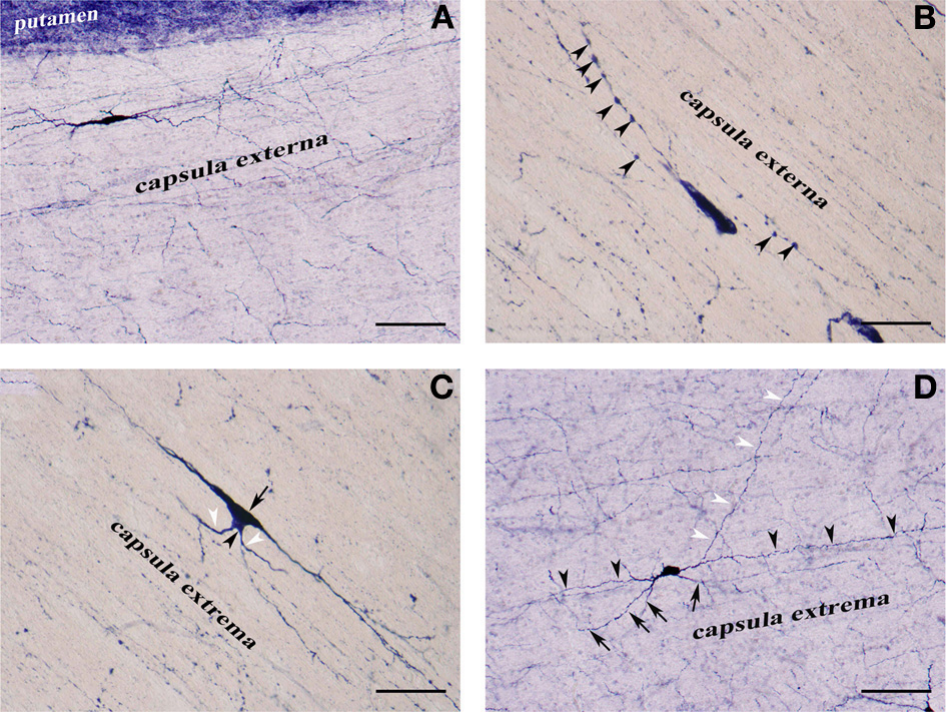
**(A)** NADPHd-positive neuron located within the extreme capsule bordering the putamen. Scale bar = 100 μm. **(B)** NADPHd-positive neuron located within the extreme capsule. Note the prominent dendritic varicosities (arrowheads). Scale bar = 40 μm. **(C)** Two NADPHd-positive neurons located within the extreme capsule, one fusiform in shape (black arrow), the second, a smaller neuron, closely abutting the first (black arrowhead). Note the short dendrites on the smaller neuron (white arrowheads). Scale bar = 40 μm. **(D)** Multipolar NADPHd-positive neuron located within the extreme capsule. Some of the secondary dendrites can be seen running parallel to the capsular fibers of the white matter (black arrowheads), while other are traversing the white matter to enter the claustrum (white arrowheads). Still other secondary dendrites are seen crossing the extreme capsule toward the putamen (black arrows). Scale bar = 100 μm.

Our results revealed a heterogeneous distribution and density of NADPHd-reactive neurons throughout the claustrum (Figure 1), often seen as clusters of 10–15 neurons (Figure 2A). Preparations through the caudal pole displayed 1–3 positive neurons per section (Figure 2B). A large number of stained cells were found in the posterior half of the rostral third of the claustrum. Conversely, positive neurons were only occasionally seen as one nears the anterior aspect of the rostral pole, and rarely in clusters. The ventral claustrum exhibited numerous NADPHd-positive neurons (Figures 2C,D), in particular, a large number (20–25 per section) within its caudal third (Figure 3A).

**Figure 4:**
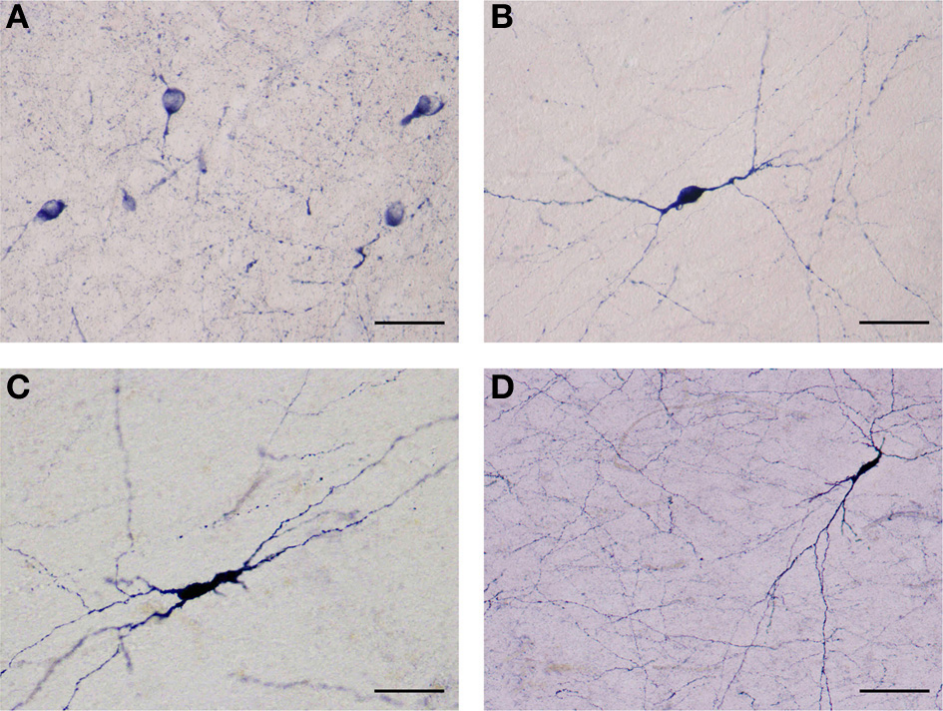
**(A)** Several NADPHd-positive neurons located in the dorsal claustrum. Reaction product can be seen filling the cytoplasm, fills the cytoplasm, while the nucleus remains stain-free. Stained puncta and fibers are also clearly visible. Scale bar = 40 μm. **(B)** Densely stained NADPHd-positive neuron with an ovoid cell body. Primary dendrites can be seen bifurcating into secondaries within a short distance, subsequently branching into tertiary dendrites coursing in many directions over a long distance. Scale bar = 40 μm. **(C)** Densely stained NADPHd-positive neuron with an irregular shaped cell body. Scale bar = 40 μm. **(D)** Large NADPHd-positive spiny neuron. The dendritic arborization is quite extensive within the claustral neuropil. Scale bar = 100 μm.

NADPHd-positive neurons were also observed within the external and extreme capsules proximal to the claustrum. As a rule, these neurons were oriented parallel to the capsular fibers (Figures 3A–C), but in some cases their projections ran perpendicular (Figure 3D). NADPHd-reaction product diffusely filled the cytoplasm (Figure 4A), while the nucleus remained stain-free. In the medial third of the claustrum a great number of neurons were lightly stained, while others resembled the intensity of a Golgi impregnation (Figures 4B–D).

A number of highly branched NADPHd-positive fibers were seen throughout the claustrum. They were readily seen to have traversed the claustrum in all of directions. This network of branching varicose fibers stood in stark contrast to the comparatively modest number of observable NADPHd-positive cells.

**Diagram 1:**
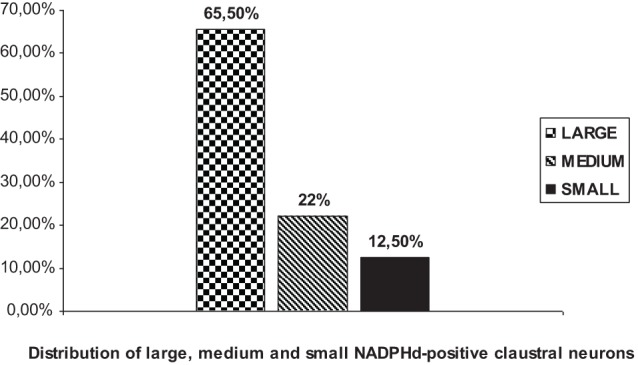
**Percent distribution of NADPHd-positive claustral neurons by diameter**.

We were able to distinguish three NADPHd-positive neuronal types and four subtypes in the human claustrum with regard to the size and shape of their perikarya and dendritic morphology. Based on our quantitative analysis, these neurons were divided into three types, as depicted in Diagram 1: large (comprising 65.5% of the total sample), medium (22%), and small (12.5%).

**Diagram 2:**
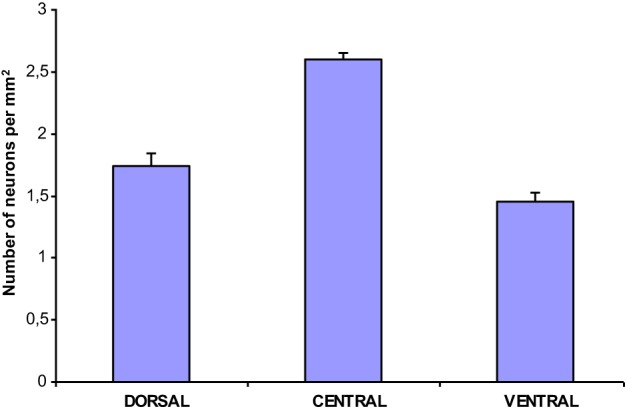
**Density of NADPHd-positive claustral neurons**.

**Diagram 3:**
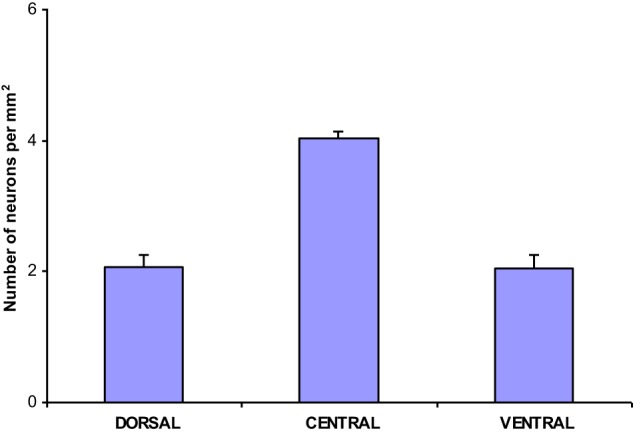
**Density of large NADPHd-positive claustral neurons**.

The population density of the 700 sampled NADPHd-positive neurons in the dorsal, ventral and central zones of claustrum (Diagram 2)—two-thirds of which were of the large cell type—showed a statistically significant difference between the dorsal and central areas (*p* < 0.001), ventral and central areas (*p* < 0.01), and dorsal and ventral areas (*p* < 0.005). The population density of large NADPHd-positive neurons was shown to be representative of the overall sampled population in two of the three comparators (Diagram 3): dorsal and central (*p* < 0.001) and ventral and central (*p* < 0.01). No statistically significant differences in population density was noted for medium and small neurons between dorsal and ventral areas (*p* < 0.05).

### Large NADPHd-positive neurons

**Figure 5:**
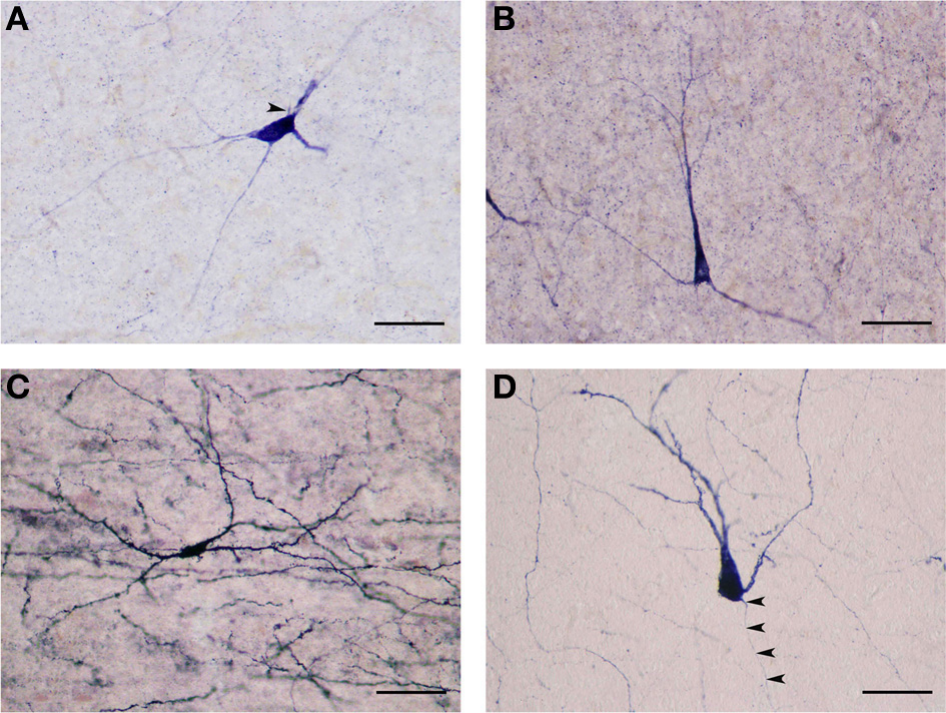
**(A)** Large NADPHd-positive neuron with a multipolar cell body. Four dendrites are seen leaving the cell body, coursing in different directions. The axon hillock can be seen emerging from the cell body, neighboring the primary dendritic trunk (arrowhead). Scale bar = 40 μm. **(B)** Large NADPHd-positive neuron with a pyramidal cell body. Note the stain-free nucleus. Scale bar = 100 μm. **(C)** Large NADPHd-positive spiny neuron with bipolar cell body and spine-covered dendrites. Scale bar = 100 μm. **(D)** Large NADPHd-positive spiny neuron with a pear-shaped cell body. Two closely situated dendrites can be seen emerging from its apex, a third leaving its base along with a thin axon initial segment (arrowheads). Scale bar = 40 μm.

Large NADPHd-positive neurons represented 65.5% of the total sampled (459/700). They ranged from 25 to 35 μm in diameter and were seen throughout the claustrum, though predominantly in its dilated rostroventral aspect (Figure 2C). Not unlike what has been seen in other species their shape varied widely, most commonly: multipolar (Figure 5A), pyramidal (Figure 5B), bipolar (Figure 5C), oval and pear (Figure 5D). Further, the large neurons could be divided into two subtypes by virtue of their dendritic morphology: spiny and aspiny.

#### Large spiny neuron subtype

**Figure 6:**
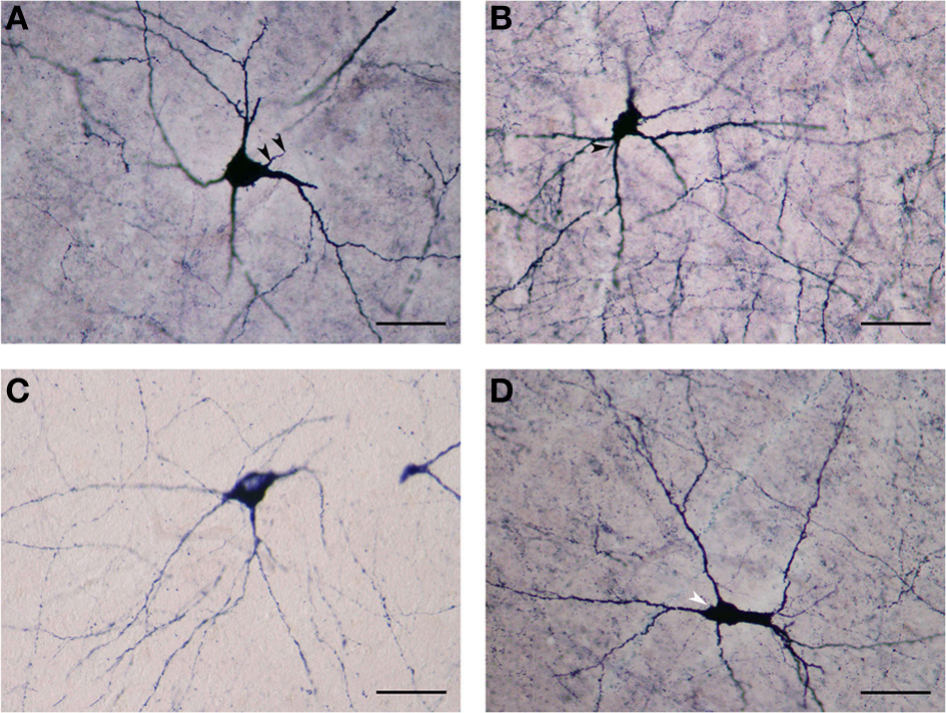
**(A)** Large NADPHd-neuron with a pear-shaped cell body and four thick primary dendrites. The axon can be seen emerging from the base of the apical dendrite (arrowheads). Secondary and tertiary dendrites are rich with spines. Scale bar = 40 μm. **(B)** Large NADPHd-positive spiny neuron with a multipolar cell body and spine-covered dendrites. The axon is seen leaving the base of one of the dendritic trunks (arrowhead). Scale bar = 40 μm. **(C)** Large NADPHd-positive neuron with a tripolar cell body and three dendrites branching into secondary and tertiary spine-covered dendrites. Scale bar = 40 μm. **(D)** Large NADPHd-positive spiny neuron with an irregular shape and five spine-covered dendrites. The axon hillock is also visible (arrowhead). Scale bar = 40 μm.

The NADPHd-positive neurons in this subtype were commonly found in the dilated rostroventral claustrum (Figure 2C), but could also be seen in its dorsal aspect. They ranged from 25 to 35 μm in diameter and displayed elliptical or multipolar perikarya, from which emanated 4–6 thick primary dendrites, each in turn producing secondary and tertiary branches covered with spines (Figures 6A,B). These secondary and tertiary dendrites took a wavy course and radiated 700–800 μm from the cell body in all directions, as well as seen crossing the capsules. In all instances, the dendrites were spiny, with axons arising from perikarya with a distinct axon hillock (Figure 6A), or from the primary dendrite as can be the case with dopaminergic neurons (Figures 6A–D). The characteristics of the dendritic tree were very much dependent upon the shape of the cell body.

#### Large aspiny neuron subtype

**Figure 7:**
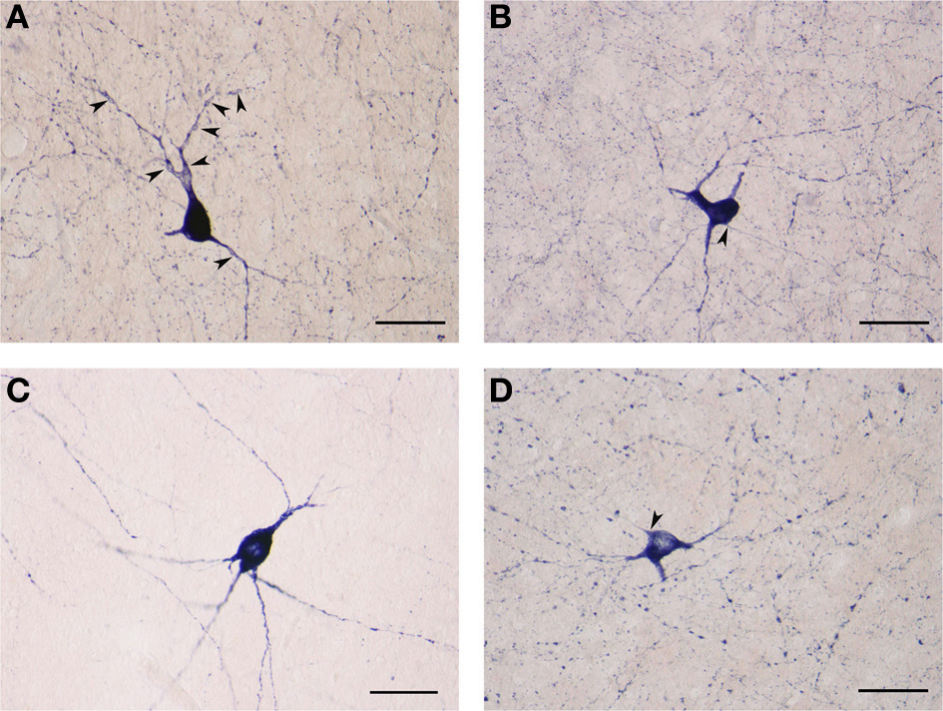
**(A)** Large NADPHd-positive aspiny neuron with a pear-shaped cell body and varicose dendrites (arrowheads). Scale bar = 40 μm. **(B)** Large NADPHd-positive neuron with an irregular cell body and aspiny dendrites. The axon is seen leaving the cell body (arrowhead). Scale bar = 40 μm. **(C)** Large NADPHd-positive aspiny neuron with varicose dendrites. Scale bar = 40 μm. **(D)** Large NADPHd-positive aspiny neuron with varicose dendrites. The axon arises from the cell body with a clearly visible hillock (arrowhead). Scale bar = 40 μm.

The NADPHd-positive neurons in this second subtype measured 25–30 μm, slightly smaller than the first. Most of these cells had a pear-shaped or irregular cell body producing a small number of far-reaching multidirectional dendrites from a cone-shaped proximal stem (Figures 7A–D). The dendritic diameter remained fairly constant along its length. The dendritic arbor bore a close resemblance to that of claustral stellate cells. The axon emerged from the cell body with a readily distinguishable axon hillock and initial segment while giving off a few thin collaterals, and was occasionally seen emanating from the trunk of the primary dendrite (Figures 7B,D).

### Medium NADPHd-positive neurons

These cells varied from 20 to 25 μm in diameter and comprised 22% (154/700) of sampled NADPHd-positive neurons. As with the large-type neurons, they too were observed throughout the claustrum. Though far fewer in number relative to the large neurons, clear differences in their somatodendritic morphology allowed for the distinction of two subtypes: spiny and aspiny.

#### Medium spiny neuron subtype

**Figure 8:**
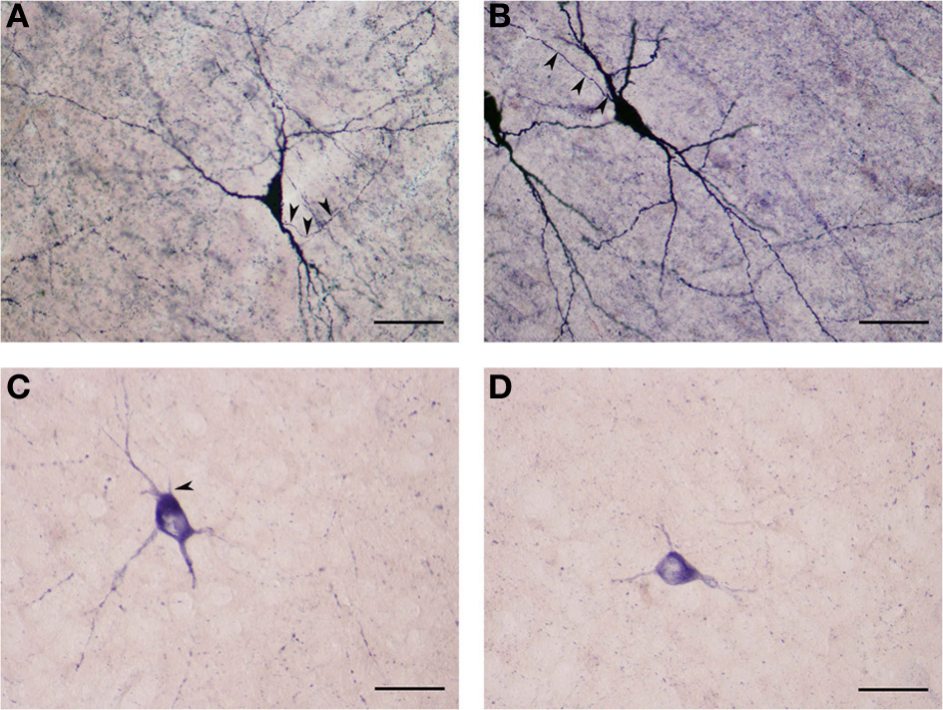
**(A)** Medium NADPHd-positive spiny neuron with a tripolar cell body and three clearly visible spine-covered dendrites. The axon is seen arising from the initial segment of the dendritic trunk (arrowheads). Scale bar = 40 μm. **(B)** Medium NADPHd-positive spiny neurons with a bipolar cell bodies. The axon is seen emerging from the dendritic trunk of one cell (arrowheads). Scale bar = 40 μm. **(C)** Medium NADPHd-positive aspiny neuron with a multipolar cell body and thin dendrites. The axon is seen leaving the cell body (arrowhead). Scale bar = 30 μm. **(D)** Medium NADPHd-positive aspiny neuron with short varicose dendrites. Scale bar = 30 μm.

These NADPHd-positive neurons displayed multipolar, bipolar or irregular cell bodies (Figures 8A–D). Their perikarya typically gave rise to 3 or 4 dendrites (on rare occasion, five). The primary dendrites bifurcated (or trifurcated) into secondary branches which often took a markedly divergent course. As a rule, the dendrites in this subclass were spiny, especially along the secondary and tertiary branches. The secondary and tertiary dendrites were wavy in form, with the most distal branches extending 400–500 μm from the cell body. The axon was noted to emanate directly from the cell body (Figures 8B,C), with a somewhat thin initial segment that could be followed for 100–200 μm, at which point the labeling abruptly ended.

#### Medium aspiny neuron subtype

**Figure 9:**
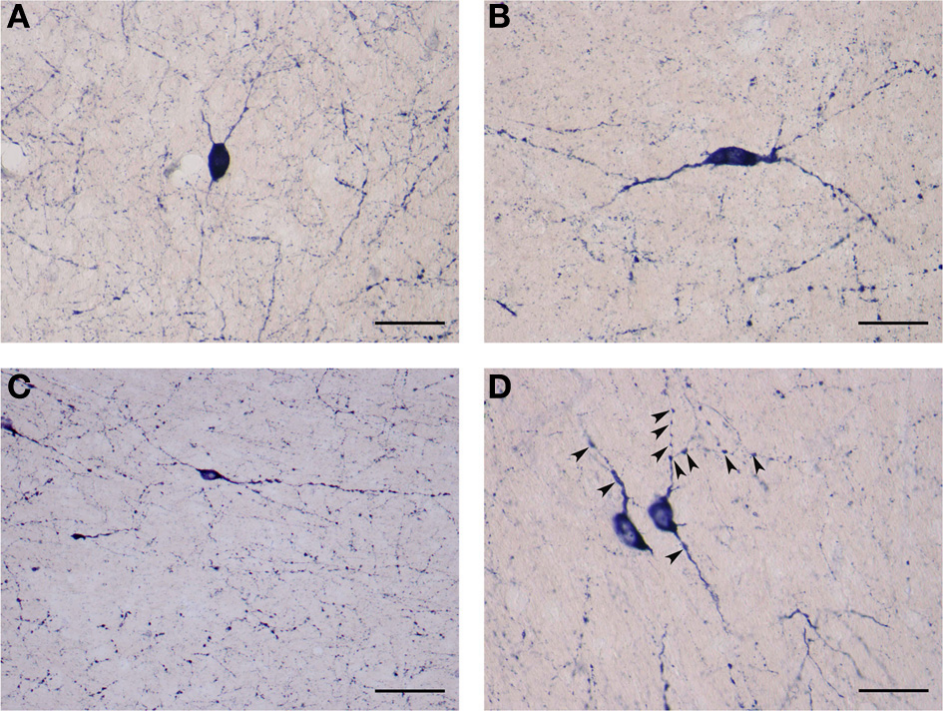
**(A)** Medium NADPHd-positive aspiny neuron, densely stained with bulbous thin dendrites. Scale bar = 40 μm. **(B)** Medium NADPHd-positive aspiny neuron with a bipolar cell body and varicose dendrites. Scale bar = 40 μm. **(C)** Small NADPHd-positive aspiny neuron with elliptical cell body and two thin varicose dendrites. Numerous stained puncta and fibers are visible. Scale bar = 100 μm. **(D)** Small NADPHd-positive aspiny neurons with thin and varicose dendrites (arrowheads). Scale bar = 40 μm.

The second subtype of medium NADPHd-positive neurons is based on shape, prominent varicosities and a paucity of dendritic spines. Multipolar, oval, irregular, or bipolar cells were observed (Figures 9A,B). Two to four considerably thin and short beaded dendrites were noted originating from the cell body, radiating in all directions. Rarely were they seen to branch more than once, while giving rise to equally fine caliber and divergent secondaries with prominent varicosities. In some cases, parts of the dendrite were dilated into large elongated bulbs (Figures 9A,B). The most distal dendritic branches could be tracked 250–400 μm from the cell body.

#### Small NADPHd-positive neurons

The third type of NADPHd-positive claustral neuron consists of small cells ranging from 14 to 20 μm in diameter, representing 12.5% (87/700) of all those sampled. Their shape was most often seen as either oval or elliptical, with an axon and 2–3 dendrites extending from the cell body (Figures 9C,D). Primary dendrites were usually thin in caliber with marked varicosities, rarely branching, and typically extending no more than 100–150 μm from the cell body, in rare instances 200–250 μm (Figure 9C). The axons were rarely seen as being stained. In principle, only the axon hillock and a short portion of the initial segment could be labeled.

## Discussion

The present study provides for the first detailed investigation of the distribution, size, characteristics, and morphology of NADPHd-positive neurons in the human claustrum. Our results confirm and extend the findings of the existence of such neurons in the human (Edelstein et al., 2012a,b), cat (Switka et al., 1994; Hinova-Palova et al., 1997), and rat (Vincent and Kimura, 1992). Generally speaking, our findings do not fully support the results of earlier investigations by Mizukawa et al. (1989) and Vincent and Kimura (1992) on the distribution of NADPHd-reactive neurons in the human claustrum. Moreover, we confirm the findings drawn by many investigators, in particular, that claustrocortical connections are distributed differently in the cortical areas (Norita, 1977; Divac, 1979; Macchi et al., 1981, 1983; Druga, 1984; Minciacchi et al., 1985; Guildin et al., 1986; Hinova-Palova, 1986; Druga et al., 1990; Dinopoulos et al., 1992). These findings have demonstrated that there is a tendency toward topographic specificity, as reported by Macchi et al. (1981; 1983), and Minciacchi et al. (1985).

In comparing the observed distribution pattern of NADPHd-reactive neurons and the existence of cellular density gradients relative to their anteroposterior and dorsoventral locations, our data confirm the investigations of Druga (1984), Druga et al. (1990), Markowitsch et al. (1984), Guildin et al. (1986), Hinova-Palova et al. (1988), and Dinopoulos et al. (1992).

Based on our analysis of seven-hundred cells sampled from across the claustrum's dorsoventral and rostrocaudal continuum, we were able to categorize three types and four subtypes of NADPHd-positive neurons based on several defining characteristics, most notably, size: large (25–35 μm), medium (20–25 μm), and small (14–20 μm).

The present results confirm our previous study (Hinova-Palova, 1986), in which we had shown that the size and morphology of the large- and medium-sized NADPHd-positive cells correspond to spiny and aspiny type II and III neurons, which we viewed as projection neurons. This supposition was also confirmed by our use of HRP injections into various auditory fields in the cat and assessing retrograde labeling in the claustrum (Hinova-Palova et al., 1988). We found a heterogeneously distributed population of labeled projection neurons, demonstrating a similarly varied pattern.

There are contradictory data on the cellular composition, size and morphology of claustral efferents. According to the classification scheme of Brand (1981) there are three types of neurons in the primate claustrum, with only one type thought to be a projection neuron. Braak and Braak (1982) described five types of neurons in the human claustrum. In our previous Golgi investigation in the cat claustrum we defined four types of projection neurons (Hinova-Palova, 1986). Using the Golgi-Cox and HRP methods, Dinopoulos et al. (1992) described three subtypes of spiny projection neurons in the hedgehog claustrum on the basis of shape and the number of primary dendrites, as well as the presence of aspiny interneurons. In addition, numerous experiments have shown that claustral projection neurons are a heterogeneous neuronal population (Hinova-Palova, 1986; Neal et al., 1986; Hinova-Palova et al., 1988; Druga et al., 1990; Claska et al., 1992; Dinopoulos et al., 1992).

A significant variety of corticoclaustral and claustrocortical circuits of the claustrum is well known. Although the claustrum has also been shown to project to various subcortical nuclei, most notably the neostriatum, zona incerta, and sensory thalamic nuclei (Druga, 1972; Sloniewski et al., 1985; Carey and Neal, 1986; Hinova-Palova, 1986), defining the characteristics of those cells which target the cortex vs. subcortical efferents remains to be determined. The somatodendritic morphology of the labeled large and medium spiny neurons we have defined in this study leads to our belief that they are projection neurons.

It is our contention that NO plays an integral role in the function and regulation of claustrocortical connections. NO is colocalized with classical neurotransmitters and neuropeptides. In this regard, somatostatin and neuropeptide Y were observed in the striatum and cerebral cortex (Vincent et al., 1983; Bredt et al., 1990; Dawson et al., 1991), in the cat claustrum (Hinova-Palova and Christova, 1988a,b) and in the human claustrum (Hinova-Palova and Braak, 1993). Edelstein et al. (2010) reported on neuropeptide Y-containing neurons in the cat claustrum, suggestive of an analogous colocalization. The third type of positive neuron we have seen in the present investigation is dispersed through the claustrum, morphologically resembling the small neurons seen in our earlier study of the cat claustrum (Hinova-Palova, 1986). Using the Golgi impregnation method we were able to divide the small neurons into two types: spiny and aspiny (Hinova-Palova, 1986). They were noted to have oval, round, or elliptical cell bodies with 3–5 markedly varicose dendrites. These cells closely resembled the small NADPHd-positive neurons characterized in the present study. As a result, we believe that it is accurate to state that the small NADPHd-positive neurons we have detailed in this study are morphologically if not functionally similar to the NOS-containing interneurons of the cerebral cortex as described by De Felipe (1993) and Rodrigo et al. (1994) in the rat.

Our findings of an extensive network of NADPHd-positive varicose and branching fibers in the claustrum prompt questions as to their etiology. The majority of these fibers represent dendrites and axons. Most likely, some of those fibers are snippets of afferents emanating from neurons in various topographically-linked areas (Hinova-Palova et al., 1984, 1988; Hinova-Palova, 1986; Druga et al., 1990). Indeed, a reasonable proportion of these fibers may well-arise from the neurons located in layer VI of cortex and the putamen which have traversed the external and extreme capsules.

Numerous investigations conducted over the past decade have led to a detailed understanding of the types of neurons which are capable of generating NO, and their distribution. NOS often colocalizes with classical neurotransmitters and neuropeptides (Vincent et al., 1986; Spike et al., 1993; Gabbot and Bacon, 1995; Rushlow et al., 1995; Feguerdo-Cardenas et al., 1996), and thus NO synthesis is often coupled to the release of these transmitters. In this regard, it is of interest to note that NOS is found in neurons using the excitatory neurotransmitter glutamate, as well as the inhibitory neurotransmitter GABA (Spike et al., 1993; Gabbot and Bacon, 1995). The colocalization of NADPHd-reactive neurons and GABAergic neurons suggests that these interneurons are inhibitory. NOS is also found in cholinergic neurons and other aminergic cells. NOS is also present in the local circuit interneurons of many brain regions (Duchemin et al., 2012). However, in some areas it is found in principal neurons with lengthy projections. In addition, NOS is found in neurosecretory cells and in some circumventricular organs (Alm et al., 1997). Thus, as with other neurotransmitters, it is difficult to generalize regarding the role of NO in the nervous system. NOS is localized in cell bodies, axons, dendrites, and nerve terminals (Edelstein et al., 2012a,b). Therefore, it is reasonable to assume that NO production may be triggered by the activation of postsynaptic calcium channels and the release of intracellular calcium stores in dendrites and cell bodies, or by the opening of voltage-gated calcium channels in nerve terminals.

The clinical relevance of NADPHd is manifest in several ways with respect to neurodegenerative diseases. Extensive studies support the contention that NADPHd-positive neurons are spared from hypoxic-ischemic insults in several disorders, including Huntington's disease (Ferrante et al., 1985) and Parkinson's disease (Hunot et al., 1996). The selective sparing of NADPHd-positive neurons has been attributed to a resistance against N-methyl-D-aspartate or quinolinate toxicity (Koh et al., 1986; Koh and Choi, 1988). A distinct subset of striatal neurons—those containing NADPHd—was shown to be selectively resistant to the degenerative process that affects the striatum in Huntington's disease (Ferrante et al., 1985, 1987). Boegman and Parent (1988) injected the tryptophan metabolite quinolinic acid unilaterally into rat cerebral cortex and striatum in order to determine whether the neurotoxin would ablate neuropeptide Y-, somatostatin-, and NADPHd-containing neurons. Following intrastriatal injections of quinolinic acid, activity of all three were absent from the injection core area. In contrast, cortical neuropeptide Y-, somatostatin- and NADPHd-containing neurons proved resistant. These results suggest that, in contrast to striatal neurons, cortical somatostatin- and neuropeptide Y- containing neurons do not express NMDA receptors. Mufson and Brandabur (1994) reported that NADPHd-containing neurons within the striatum are spared in patients with Parkinson's disease and Alzheimer's disease. However, a number of these neurons in both diseases appeared shrunken or bulbous with shortened dendritic processes. Tao et al. (1999) studied the involvement of NADPHd-containing neurons in the cortex, subcortical white matter and striatum of Alzheimer's disease patients. Despite slight morphological changes in the cortex of the Alzheimer's patients, they found no significant difference in the number of NADPHd-positive neurons in either the cortex or the striatum when compared with the cortex of similarly aged controls. Their results provide further evidence for a selective preservation of NADPHd neurons in Alzheimer's disease. Freire et al. (2007) used NADPHd histochemistry to investigate the effects of mercury intoxication on the structure of the posteromedial barrel subfield in the primary somatosensory cortex of adult rats. It was found that NADPHd reactivity in the neuropil of barrel fields was drastically reduced in experimental animals, suggesting that the synthesis and transport of NOS can be altered during acute mercury intoxication. However, the cell bodies and dendrites of barrel neurons, also strongly reactive to the enzyme, were spared from mercury's deleterious effects.

This is the first detailed investigation of the light- and electron-microscopic features of the NADPHd-containing neurons and fibers in the human claustrum, a subcortical nucleus known to have reciprocal connectivity with nearly all cortical areas. It is hoped that a deeper understanding of the relationship between NADPHd and the sequelae of neurodegenerative disorders, as well as of NOS distribution and reasons for its colocalization with other neurotransmitters and neuropeptides, will help to better define the role of NO in the context of claustral function, the interplay between the claustrum and cerebral cortex, and of the nervous system proper.

### Conflict of interest statement

The authors declare that the research was conducted in the absence of any commercial or financial relationships that could be construed as a potential conflict of interest.
